# Transumbilical laparo-endoscopic single site surgery for adrenal cortical adenoma inducing primary aldosteronism: initial experience

**DOI:** 10.1186/1756-0500-4-364

**Published:** 2011-09-24

**Authors:** Akira Miyajima, Takahiro Maeda, Masanori Hasegawa, Toshikazu Takeda, Masaru Ishida, Takeo Kosaka, Eiji Kikuchi, Ken Nakagawa, Mototsugu Oya

**Affiliations:** 1 Keio University School of Medicine, Department of Urology, 35 Shinanomachi, Shinjuku, Tokyo 160-8582, Japan

## Abstract

**Background:**

We have started using laparo-endoscopic single-site surgery (LESS) in urologic surgery, although its use has not gained momentum due to its level of difficulty. We here report our initial experience with transumbilical LESS for adrenal cortical adenoma by using a single port with a multichannel cannula (SILS port) and bent laparoscopic instrumentation.

**Findings:**

A multichannel port (SILS port), bent laparoscopic instrument (Roticulator Endo Mini-Shears) and Opti4 laparoscopic electrodes were used in all cases. The intraperitoneal space was approached through the umbilicus. The SILS port was placed through a 2 cm incision at the inner edge of the umbilicus. A 5 mm flexible laparoscope was introduced to keep the laparoscope outside, and surgical specimens were extracted using an Endocatch bag. In addition, as a case control study, we compared perioperative data of LESS adrenalectomy (LESS-A) with that of conventional laparoscopic adrenalectomy (LA). We performed transumbilical LESS-A for adrenal cortical adenoma in 12 cases, beginning in December, 2009. All procedures were successfully completed, with only one incision through the umbilicus, and without conversion to a standard laparoscopic approach. Mean operative time for LESS-A was 121.2 ± 7.8 min, which was slightly longer than LA (110.2 ± 7.3 min). For right adrenal tumors, we used a miniport (2 mm port) in addition to a SILS port, and were able to successfully perform adrenalectomy "with no visible scaring". Tumor laterality and patient BMI did not affect surgical morbidity in these procedures. Moreover, there was no significant difference between LESS-A and LA in blood loss, analgesic requirement, hospital stay, and scar satisfaction.

**Conclusions:**

The transumbilical approach in LESS for adrenalectomy is safe and feasible and also improves cosmetic outcome compared with standard laparoscopic procedures. Improvements in surgical devices may aid the further development of this approach.

## Background

Laparoscopic surgery is a well-established alternative to open surgery across many disciplines. Although its magnitude of impact varies by procedure, in general, the benefits of laparoscopy on postoperative pain, cosmesis, hospital stay, and convalescence are widely recognized. Current efforts are aimed at further reducing the morbidity associated with minimally invasive surgery. Current laparoscopic techniques involve the use of three to six small skin incisions, depending on the complexity of the procedure. This step induces temporary incisional pain or muscle spasms.

The umbilicus is an obliterated embryonic (E) orifice, through which portal access can be obtained with concealment of the incisional scar. Single-port laparoscopy through the umbilicus has been termed E-NOTES, and offers an exciting opportunity for performance of major laparoscopic surgery with no visible scar. As such, E-NOTES could potentially serve as a bridge between conventional multiport laparoscopy and NOTES[1]. Laparoscopic single-site surgery (LESS) has been reported for cholecystectomy[2], appendectomy[3], and urologic surgery[4], although its use has not gained widespread momentum due to its high degree of difficulty. We present here our initial experience with LESS at our institution, especially using a transumbilical approach for adrenalectomy.

## Implementation

This is a retrospective case control study comparing 12 laparo-endoscopic single-site surgery adrenalectomies (LESS-As) performed between December 2009 and September 2010 to 24 conventional laparoscopic adrenalectomies (LAs) performed between April 2006 and August 2010. Since December 2009 at our institution, transumbilical LESS-A has been performed in 12 patients (4 male, 8 female, mean age: 57.4 ± 3.5 y/o), who had cortical adenomas inducing primary aldosteronism. From April 2006 to September 2010, 94 conventional LAs were performed at out institution. From this cohort, we selected 24 patients (18 male, 6 female, mean age: 55.9 ± 2.0 y/o) to serve as a control group for this study. These patients were specifically matched in a 2:1 ratio to index LESS-A cases with respect to disease, patient BMI and tumor laterality. No consideration or analysis of operative parameters and outcomes was made until this group was definitely selected as the best comparison cohort based on preoperative variables only. These 24 patients had undergone LA for primary aldosteronism, with the first being performed on April 1, 2006. All procedures were performed by the same surgical team at our hospital. Operative time, blood loss, analgesic requirement, length of stay, postoperative complications, and final pathology were recorded. We statistically compared these two groups in terms of perioperative data and patient scar satisfaction level, which was measured using a 0-10 scale after surgery[5]. Data were collected prospectively following Institutional Review Board approval. All procedures were performed by a single surgeon (A.M.).

**Figure 1:**
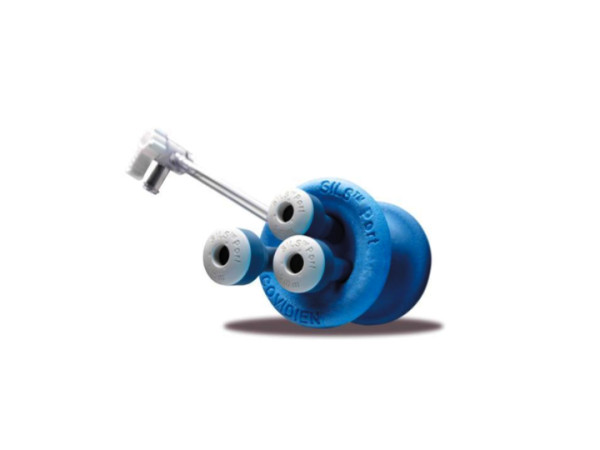
**SILS port basically provides three ports for 5-12 mm laparoscopic bent instruments and a flexible laparoscope**. One port can be compatible from 5mm to 12 mm.

**Figure 2:**
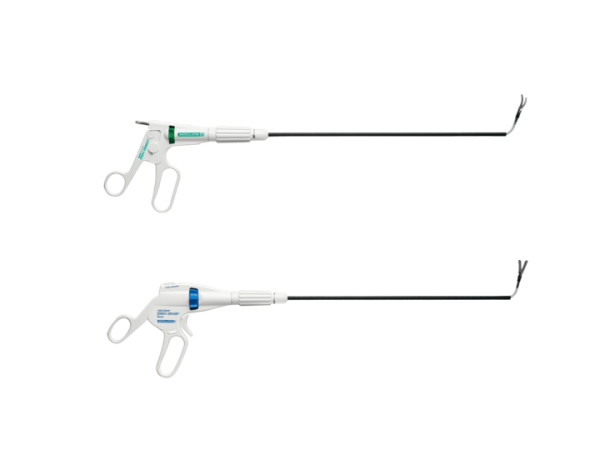
**Bent laparoscopic instruments with rotational mid-shaft and instrument tip, and curved shaft can be changed to desired orientation using the knob on the handle**.

**Figure 3:**
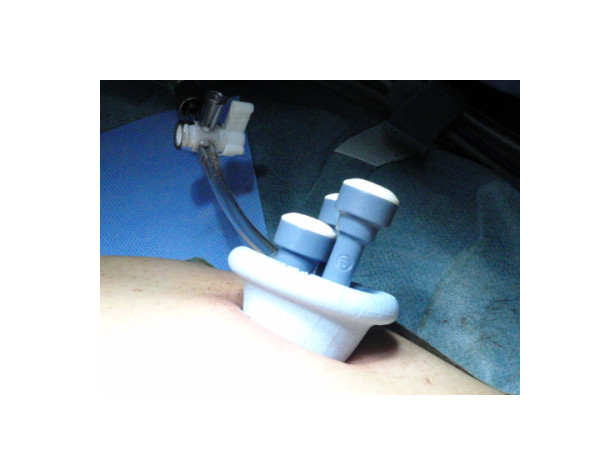
**Intraoperative photograph of SILS port placed in umbilicus**.

**Figure 4:**
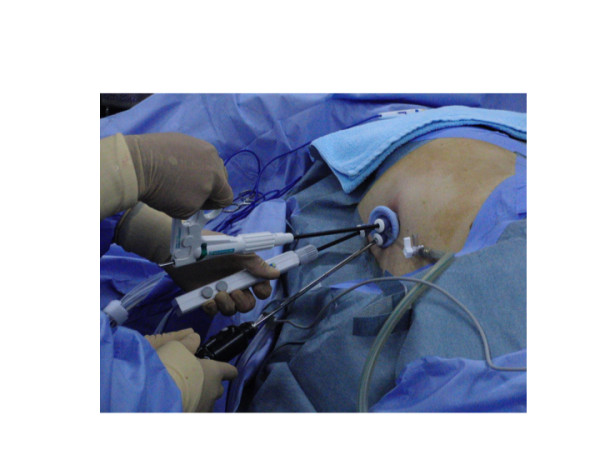
**Instruments were inserted through SILS port**.

**Figure 5:**
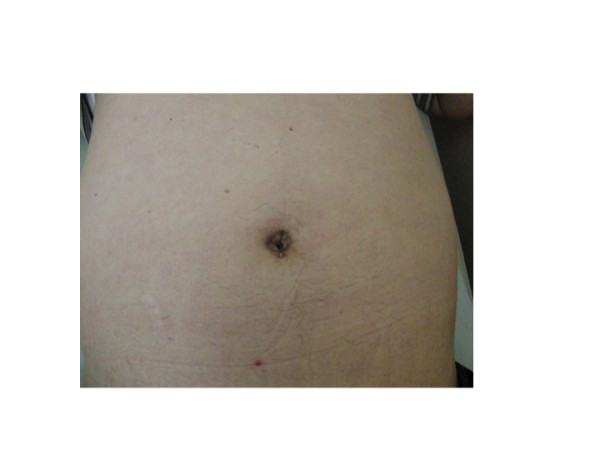
**Postoperative photograph of skin incision of a patient who underwent right adrenalectomy**.

**Figure 6:**
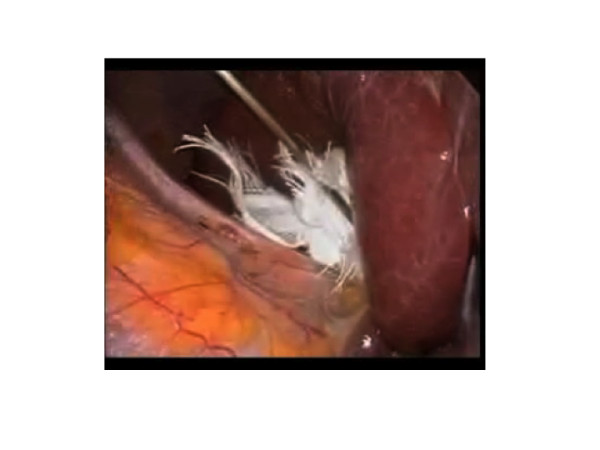
**The liver is lifted using gauze and a miniport forceps. 4b Longer hook for Opti4 laparoscopic electrode**.

The multichannel port (SILS port, Figure 1), bent laparoscopic instrument (Roticulator Endo Mini-Shears, Figure 2) and Opti4 laparoscopic electrodes were supplied by Covidien (Mansfield, USA). Ultrasonic scalpel (SonoSurg) was supplied by Olympus Surgical (Tokyo Japan). In all cases of LESS-A, we approached the intraperitoneal space through the umbilicus. The SILS port was placed through a 2 cm incision at the inner edge of the umbilicus (Figure 3). The anterior rectus fascia was sharply incised, and four corner fascial stay sutures were placed. A 5 mm flexible laparoscope (Olympus Surgical, Tokyo, Japan) was introduced to keep the laparoscope outside the port in a location away from the surgeon's instruments to avoid instrument contact and maximize surgical movement. Bent instruments were required to create the operative angle because the insertion points were quite close to each other (Figure 4). The instrument in the right hand was placed on the left side of the screen, and the left hand instrument was placed on the right side of the screen. They were articulated in opposite directions. Intraoperative dissection and vessel cutting were performed in the usual fashion in all cases. No cases needed extension of the umbilical incision to remove adrenal specimens using an Endocatch bag (Covidien, Mansfield, USA). Hemostasis was carefully maintained and no drainage tubes were left in any of the cases. The fascial incisions were closed with absorbable suture, and the umbilicus was restored with absorbable cutaneous stitches to its original state (Figure 5). For right-sided disease, which we experienced in 5 cases of this series, the liver had to be lifted. In such cases, we inserted a 2mm Miniport (Covidien, Mansfield, USA), from the lateral side of the abdomen. Although 2mm port insertion appears to be scar-less, a 2 mm forceps can be traumatic for the liver. To avoid traumatic procedure, we used a small gauze or Securea sponge (Hogy, Tokyo) as cushioning to lift the liver (Figure 6). In all cases of LA, we placed 3 or 4 ports into the intraperitoneal space in an usual fashion, and performed adrenalectomy by using rigid straight laparoscopic instruments and a rigid laparoscope under pneumoperitoneum.

Groups were compared using the chi-square test and Mann-Whitney U-test for categorical and continuous variables, respectively. Statistical significance was set at *p *< 0.05, and all reported *p *values are two-sided. These analyses were performed using SPSS.

## Results and Discussion

All cases of LESS-A were successfully completed without any intraoperative complication and any conversion to conventional LA. The patient background and early perioperative data for 12 patients are shown in Table 1. The mean operative time was not significantly different for left side tumors (118.4 ± 13.1 min, n = 5) and right side tumors (122.7 ± 29.3 min, n = 7) in LESS-A while neither BMI nor tumor size was different between the two sides. In order to investigate the difference between LESS-A and conventional LA (18 male and 6 female; mean age: 55.9 ± 2.0; BMI: 25.0 ± 0.7; tumor size: 14.8 ± 1.4 mm; n = 24), we statistically compared these two groups in terms of perioperative data (Table 2). Compared with LA (110.2 ± 7.3 min), LESS-A appeared to require a longer operation time (121.2 ± 7.8 min), although the difference was not statistically significant. In addition, there were no differences in blood loss (not detectable in both groups), analgesic requirement (flurbiprofen axetil 2.9 ± 0.5 mg vs. 2.9 ± 0.5 mg, *p *= 0.501), and length of stay (5.2 ± 0.3 days vs. 6.1 ± 1.0 days, *p *= 0.687). There were no postoperative complications in either group. All cases started oral intake and ambulation on POD 1. Furthermore, we investigated the level of patient scar satisfaction, and scar satisfaction was also comparable in both groups (LESS: 9.67 ± 0.33; LA: 9.47 ± 0.28, *p *= 0.889).

**Table 1:** Patient background and perioperative data

No.	Age	Gender	BMI	Laterality	Tumor size (mm)	Operating time (min)	Incision length (cm)	Analgesic requirement (mg)	Hospital stay (POD)
1	59	F	19.1	L	8	99	2.5	200	6

2	29	F	22.3	L	25	95	2.5	150	5

3	61	M	30.6	R	10	160	2.5	50	5

4	49	F	19.8	R	20	91	2.5	200	5

5	67	F	23.3	L	25	133	2.5	100	6

6	61	M	29.7	R	40	163	2.5	200	5

7	41	M	27.4	R	12	120	2.5	150	4

8	67	F	33.8	R	52	152	3	100	5

9	59	F	27.4	L	10	130	2.5	250	5

10	69	M	22.4	R	10	120	2.5	100	5

11	56	F	20.9	R	12	86	2.5	50	4

12	70	F	26.3	L	25	106	2.5	0	4

Mean	57		25.2		20.8	121.2	2.5	146	4.9

Analgesic requirement is indicated by the dose of flurbiprofen axetil.

In all cases, blood loss was not detectable.

**Table 2:** Comparison of perioperative data between LESS-A and LA

	LESS-A	Conventional LA	P Value
Total no.	12	24	
Mean age	57.4 ± 3.5	55.9 ± 2.0	0.402
Male/Female ratio	4(33.3%)/8(66.7%)	18(75.0%)/6(25.0%)	0.040
BMI	25.2 ± 1.3	25.0 ± 0.7	0.893
Left/Right side ratio	5(41.7%)/7(58.3%)	10(41.7%)/14(58.3%)	0.999
Tumor Size(mm)	20.8 ± 4.0	14.8 ± 1.4	0.306
Operative time(minute)	121.2 ± 7.8	110.2 ± 7.3	0.347
Estimated blood loss	N.D	N.D	
Analgestic requirement	2.9 ± 0.5	2.9 ± 0.5	0.501
Hospital stay (POD)	5.2 ± 0.3	6.1 ± 1.0	0.687

Prior to the adoption of a transumbilical approach, a pararectal incision was made in one case since it appeared to be much safer and easier to reach the target organ and vessels compared with the transumbilical approach. We confirmed the safety and feasibility of LESS, and began to use the transumbilical approach. Umbilical access does not add new risks, and appears to yield an operative view which is the same as that in standard laparoscopic surgery for the adrenal gland and kidney when using a flexible laparoscope. Conventional laparoscopic surgery requires blunt insertion of some ports, resulting in temporary incisional pain or muscle spasms[6]. Moreover, the use of only one incision within the umbilicus renders selected transperitoneal procedures scarless, and the results of a cost analysis indicated that LESS-A and LA are comparable in terms of cost. Although patient scar satisfaction was comparable between LESS-A and LA in the present study, the cosmetic outcome of LESS can definitely be improved compared with conventional laparoscopic procedures.

Two of our adrenalectomy patients had a high BMI (30.2; 33.8) and although they had much fat tissue, resulting in difficulty with visualization, the procedures were successfully performed within 3 hours. In addition, the transumbilical approach required longer hooks for the laparoscopic electrodes (Figure 4b). Although it has been reported that a high BMI may be a contraindication for LESS, we believe that such cases will become treatable with LESS as its instrumentation improves [7].

In LESS, avoiding contact interference between the operative instruments and the laparoscope is essential for maintaining adequate pneumoperitoneum and reducing operative stress. Therefore, coordination between the operating surgeon and the laparoscope assistant are vital for this procedure, since every single movement of one affects the other. The average surgical time in the LESS group appeared to be longer than in the conventional LA group. We attempted to identify the technical differences between these two methods. The difficulties in LESS surgery mainly arise from the "sword fighting" of the instruments, and this "fighting" can be reduced by using bent instruments and a cross-over technique. However, the angles of the bent instruments need to be adjusted, maneuvers which require a considerable amount of time. In endoscopic surgery, one hand performs dissection and the other hand performs traction, and it is thus necessary to coordinate these bimanual motions. Even though bent instruments have been introduced, "sword fighting" remains a concern in LESS surgery. "Sword fighting" occurs because two laparoscopic instruments and a laparoscope are introduced through the same incision. Each instrument can come into contact and interfere with the other 2 instruments in a fashion that resembles sword fighting, even though the instruments are flexible or bent-neck. Therefore, target tissue or vessels can not be easily reached.

In the early phase of our experience with LESS adrenalectomy, we spent quite a bit of time doing one-handed manipulations in order to avoid sword fighting, however, the amount of time spent has gradually decreased as the number of procedures performed has increased[8]. Because the distance from the port to the tissue in the transumbilical approach is longer than in the conventional laparoscopic approach, the approach is from a more tangential direction in LESS surgery. The different angle of approach of the instrument feels different to that of conventional laparoscopic surgery, and it is difficult to approach the target tissue in a straight-forward direction. Thus, we tend to grasp the second- or third-best site. This may contribute to an increase in tissue re-grasping due to the inadequate or insufficient counter-traction. Furthermore, because the gripping power of the Roticulator was not strong enough to keep grasping the tissue, the instruments should be further improved so that the tissue does not slip away from the Roticulator.

The inclusion criteria for LESS-A are still controversial. A large tumor (>10 cm) or an invasive tumor should be approached by LA, however, we have not experienced such cases, and some cases with severe adhesion around the target organ or unpredictable hemorrhaging may require additional port placement. Although we performed LESS-A for most adrenal tumors (PA), a prospective and randomized large series of LESS-A will most likely be required in order to determine definite indications for LESS-A.

All procedures were successfully performed in a reasonably time-efficient fashion, even in this initial experience with this new technique. We believe that modification of the instruments and new improvements, including suturing devices, which enable the surgeon to approach the target organ from any angle, will make this procedure more clinically feasible.

## Conclusions

The transumbilical approach in LESS for adrenalectomy is safe and feasible and also improves cosmetic outcome compared with standard laparoscopic procedures. Improvements in surgical devices may aid the further development of this approach.

## Abbreviations

LESS: Laparo Endoscopic Single Site Surgery

LESS-A: LESS Adrenalectomy

LA: conventional Laparoscopic Adrenalectomy

NOTES: Natural Orifice Translumenal Endoscopic Surgery

BMI: Body Mass Index

POD: Post Operative Day

PA: Primary Aldosteronism

SILS: Single Incision Laparoscopic Surgery

## Competing interests

The authors declare that they have no competing interests.

## Authors' contributions

AM drafted the first manuscript. TM and MI helped to draft the manuscript and analyze data statistically. MH, TT, TK, EK and KN cared for the patient. MO is the chair of this department. All authors reviewed the report and approved the final version of the manuscript.
